# Antimicrobial Resistance and Biofilm Formation in Bacterial Species Isolated from a Veterinary Hospital

**DOI:** 10.3390/pathogens14090845

**Published:** 2025-08-24

**Authors:** Vanessa Bridi, Débora Pereira Gomes do Prado, Stéfanne Rodrigues Rezende Ferreira, Carolina Pedrosa Pedretti, Edmar Gonçalves Pereira Filho, Wagner Gouvêa dos Santos, Hanstter Hallison Alves Rezende

**Affiliations:** 1 Laboratory of Bacteriology and Micology, Biomedicine Course, Institute of Health Sciences, Jatobá Campus, Federal University of Jataí, Jataí 75801-615, Goiás, Brazil; vanessabridi@hotmail.com (V.B.); deboraprado@discente.ufj.edu.br (D.P.G.d.P.); stefannerezende@discente.ufj.edu.br (S.R.R.F.); pedretti.carolina@discente.ufj.edu.br (C.P.P.); edmar.filho@discente.ufj.edu.br (E.G.P.F.); 2 Genetics and Molecular Biology Laboratory, Biomedicine Course, Institute of Health Sciences, Jatobá Campus, Federal University of Jataí, Jataí 75801-615, Goiás, Brazil; wagner_santos@ufj.edu.br

**Keywords:** pathogenic microorganisms, biofilms, veterinary environments, resistance

## Abstract

Micro-organisms are abundant in nature and can also be found in hospital settings, causing high rates of infections. This study aimed to identify bacteria isolated from a veterinary hospital, as well as to perform antimicrobial susceptibility testing using the disk diffusion method (Kirby–Bauer), biofilm production tests using 96-well polystyrene microtiter plates and crystal violet dye, and genetic analysis of the *ica* operon of *Staphylococcus* isolates. Three collections were made from eleven surfaces and objects in the hospital’s non-critical areas (general areas) and critical areas (surgical center), totaling thirty-three samples. A total of 66 different bacterial isolates were obtained, with 77% (51/66) Gram-positive and 23% (29/66) Gram-negative. Resistance profiles were found for multidrug-resistance (MDR), methicillin-resistant *Staphylococcus aureus* (MRSA), methicillin-resistant *Staphylococcus epidermidis* (MRSE), and other unidentified species of methicillin-resistant coagulase-negative (MRCNS) and extended-spectrum beta-lactamase (ESBL), as well as biofilm production rates of 57% (38/66) of the isolates. Analysis of the operon genes for *Staphylococcus* sp. showed divergence in some samples when compared to the phenotypic test performed. In summary, there is a high presence of micro-organisms with resistance and virulence factors spread throughout the various areas of the veterinary hospital.

## 1. Introduction

Hospital environments are considered important reservoirs of micro-organisms, playing a significant role in the selection and dissemination of multidrug-resistant pathogens, and leading to cases of secondary cross-infections [1,2]. In hospital environments and veterinary practices, the intense and constant movement of people and animals associated with animal health immunosuppression and risk factors such as increased hospital stays, high incidence of invasive procedures, and excessive and inadequate use of antimicrobial therapies contribute to the incidence of healthcare-associated infections (HAIs) [3,4].

The rapid spread of micro-organisms and the high capacity to produce virulence factors lead to an increase in antimicrobial resistance rates and the formation of biofilms, making the treatment of infections even more difficult [5]. Biofilms are structured and complex bacterial communities, wrapped in a self-producing polymeric matrix, which is composed of proteins, lipids, carbohydrates, extracellular nucleic acids (eRNA and eDNA), and extracellular polymeric substance (EPS) [6,7,8]. The intense bacterial proliferation and interaction provides genetic exchange between different bacterial strains, ensuring survival and conferring resistance to antimicrobials and, disinfectants, and against mechanisms of the host’s immune response [9].

The process of bacterial biofilm formation involves four main stages, which include initial reversible adhesion, irreversible adhesion, formation of microcolonies, and maturation and dispersion of persister cells [10,11]. This process is genetically regulated by specific genes for the coding of adhesion proteins [12]. For the species of *Staphylococcus* sp., *ica* operons (*ica* ADCB and gene *ica* R) responsible for the production of polysaccharide intercellular adhesin (PIA), the homologous biofilm-associated protein (Bhp), and the accumulation-associated protein (Aap) are the most important [13,14]. In addition to this, the communication system between cells, quorum sensing, plays an important role in the production of autoinducers and accessory gene regulators such as the *agr* locus (*agr* I, II and III) [12,13].

The obstacles in this issue, in addition causing severe infections with permanent consequences and even the death of the animal, lead to the possibility of social and psychological repercussions for the guardians, with high hospital costs, traumas, and loss of credibility of the team and the institution [15]. Concomitant to this, there is the ability of transmission of these multiresistant bacterial infections from animals to their owners, professionals, employees and, in teaching hospitals, to academics and residents in the area, which can cause outbreaks and acquire zoonotic potential, raising concerns at the level of One Health [3,16,17]. Furthermore, studies in veterinary environments and epidemiological data regarding bacterial contamination are still scarce and under-reported, with the topic being little discussed [18].

The aim of this study was to isolate and identify bacteria from surfaces and objects in the general areas and in the operating room of a veterinary hospital, as well as to evaluate the antimicrobial resistance profile, the biofilm formation capacity, and the presence of *ica* operon genes in isolated bacteria.

## 2. Materials and Methods

### 2.1. Study Design

Samples were collected from 11 surfaces and/or objects at a veterinary teaching hospital in the Southwest Region of Goiás, totaling 33 samples. The objects and surfaces were selected based on the literature research on the main potential locations for harboring micro-organisms in hospital environment. After analyzing several studies, the sites were selected. Collection from non-critical areas was conducted in August 2023. Samples from the critical areas were collected in September 2023 (first collection) and December 2023 (second collection).

### 2.2. Collection and Transportation of Samples

Sample collection was divided into two distinct environments: non-critical areas and critical areas (surgical suite), with one and two collection moments, respectively, totaling three collection moments (33 samples). The first collection covered non-critical areas: reception desk, offices, isolation area, hospitalization area, infectious diseases area, and pre-operative areas of the hospital. The other two collections were carried out exclusively in the two operating room areas of the hospital surgical suite. The collections were carried out with the use of sterile swabs soaked in 0.9% saline, which were passed over the objects and surfaces and then placed in identified test tubes containing brain heart infusion (BHI) (OXOID^®^, Inc., São Paulo, Brazil) broth, placed on shelves in a thermal box and transported to the bacteriology and mycology laboratory, remaining incubated for 24 h in a BOD incubator at a temperature of 35 ± 2 °C, under aerobic conditions.

### 2.3. Sample Processing and Identification

Samples positive for bacterial growth (turbidity of the BHI broth) were plated on blood agar—BA (HIMEDIA^®^, Inc., Mumbai, India) and incubated for a period of 24 h in a BOD incubator at a temperature of 35 ± 2 °C (95 °F + 35.5 °F) in an aerobic atmosphere. After the incubation time, the samples were analyzed for the diversity of colonies grown as well as their hemolysis pattern. Plates with distinct colonies were transferred to new BA plates for these colonies to be purified. Then, an isolated colony from each sample was subjected to the Gram-staining technique (NewProv Kit^®^, Inc., Pinhais, Brazil) and thus seeded on specific agar and incubated for 24 h. Subsequently, phenotypic identification tests through conventional biochemical tests were performed: fermentation of Salted Mannitol agar (HIMEDIA^®^, Inc., Mumbai, India), catalase, coagulase, DNAse, polymyxin B, and novobiocin test for Gram-positive cocci bacteria (catalase positive); catalase, optochin, bacitracin, bile-esculin test and NaCl tolerance test for Gram-positive cocci bacteria (catalase negative); catalase test and Gram technique for Gram-positive bacilli; and for Gram-negative, lactose fermentation in MacConkey agar (HIMEDIA^®^, Inc., Mumbai, India), oxidase, and rugai tests (Newprov^®^, Inc., Pinhais, Brazil) [19].

### 2.4. Antimicrobial Susceptibility Testing

Antimicrobial susceptibility testing (AST) was performed using the disk diffusion method (Kirby–Bauer). A bacterial suspension was prepared with 0.9% sterile saline in turbidity of 1.5 × 10^8^ CFU/mL (0.5 on the McFarland scale), and then seeded in a Petri dish (140 × 15 mm) with Mueller Hinton agar (HIMEDIA^®^, Inc., Mumbai, India); the antimicrobials corresponding to each isolated strain were deposited and incubated in a BOD incubator at 35 ± 2 °C for 18 to 24 h. The interpretation of the inhibition halos and the selection of antimicrobials were carried out according to parameters recommended by BrCAST [20]. For *Staphylococcus* sp., the following antimicrobials were tested: penicillin (10 µg), oxacillin (01^2^ µg), cefoxitin (30 µg), ciprofloxacin (5 µg), levofloxacin (5 µg), gentamicin (10 µg), amikacin (30 µg), erythromycin (15 µg), clindamycin (2 µg), tetracycline (30 µg), nitrofururantoin (300 µg), rifampicin (5 µg), and sulfametaxazole/trimethoprim (25 µg). MRS, MRSE, and MRCNS phenotypes were detected through oxacillin resistance. For *Enterococcus*, the following were tested: penicillin (10 µg), ampicillin (10 µg), vancomycin (30 µg), ciprofloxacin (5 µg), levofloxacin (5 µg), rifampicin (5 µg), chloramphenicol (30 µg), and nitrofururantoin (300 µg). For *Bacillus* sp., the following were tested: clindamycin (17 µg), erythromycin (15 µg), imipenem (10 µg), meropenem (10 µg), vancomycin (5 µg), ciprofloxacin (5 µg), norfloxacin (10 µg), and levofloxacin (5 µg). For Enterobacteriaceae, ampicillin (10 µg), amoxicillin-clavulanic acid (30 µg), cefepime (30 µg), cefotaxime (30 µg), ceftazidime (30 µg), ceftriaxone (30 µg), imipenem (10 µg), meropenem (10 µg), aztreonam (30 µg), ciprofloxacin (5 µg), amikacin (30 µg), gentamicin (10 µg), and fosfomycin (200 µg) were tested. The phenotype of possible extended-spectrum beta-lactamase (ESBL) Enterobacteriaceae was identified through the antimicrobials amoxicillin-clavulanic acid, cefepime, cefotaxime, ceftazidime, and aztreonam, which were positioned in a cross-shaped manner to observe the formation of an increase or distortion in the diameter of the antimicrobials and the formation of an extra synergistic zone between the disks (ghost zone). The only species that did not show resistance to any antimicrobial was the *Acinetobacter* sp. isolate, which was tested for the following antimicrobials: tricarcillin/clavulanic acid (85 µg), imipenem (10 µg), meropenem (10 µg), ciprofloxaxin (0.5 µg), amikacin (30 µg), gentamicin (10 µg), and levofloxaxin (0.5 µg). All antibiotics used were from the Laborclin^®^ (Inc., Pinhais, Brazil) brand. Antimicrobial resistance profiles were defined according to criteria established by international consensus. The strains were classified as multidrug-resistant (MDR)—present acquired resistance to at least one antimicrobial belonging to three or more distinct classes, extensively drug-resistant (XDR)—in isolates with resistance to all agents tested, except those belonging to a maximum of two therapeutic classes; that is, it remains susceptible to only one or two classes and is pandrug-resistant (PDR), which corresponds to complete resistance, with no detectable susceptibility to any agent tested, covering all available antimicrobial classes [21].

### 2.5. Biofilm Production

To perform this test, an isolated colony of each identified strain was transferred to test tubes containing 2 to 3 mL of BHI broth and incubated in a BOD incubator at 35 ± 2 °C for 24 h. Subsequently, the test was assembled in sterile 96-well polystyrene microtiter plates and deposited in all wells of the plate, 198 μL of tryptic soy broth—TSB (OXOID^®^, Inc., São Paulo, Brazil); supplemented with 1% glucose (MERCK^®^, Inc., Darmstadt, na Alemanha) and 2 μL of BHI + strain, totaling 200 μL per well. The positive control used was the ATCC 0027 strain *Pseudomonas aeruginosa* and the negative control was TSB broth + 1% glucose. All samples and controls were tested in triplicate. The plate was covered and taken to the BOD incubator, 35 ± 2 °C, for 48 h and the reading continued in four different steps: plate washing 3 times with 300 uL of 0.9% sterile saline, heat fixation in an incubator at 60 °C (1 h), staining with 150 uL of crystal violet at 2% (15 min), resolubilization with 150 uL of 95% alcohol (30 min), and, finally, measurement of the results in a microtiter plate reader (Thermo Plate^®^, Inc., Tóquio, Japão) at 550 nm. The averages of the triplicates were calculated and compared with the average of the negative control, performed using the cutoff point (Cut off—ODc) according to the formula: ODc = (Average OD-) + (3 x sd). The strains were classified as non-biofilm producers, and weak, moderate, and strong biofilm producers. The values were interpreted as described by Stepanovic et al. [22]: Samples considered non-biofilm producers were those whose optical density (OD) was less than, or equal to, the OD of the control sample (ODcontrol). Samples with an OD greater than (up to 2 times) the ODcontrol were classified as weak biofilm producers (+ or 1). Those with an OD between two and four times the ODcontrol were classified as moderate biofilm producers (++ or 2). Samples with an OD greater than four times the ODcontrol were classified as strong biofilm producers (+++ or 3). [22].

### 2.6. DNA Extraction from Isolates

DNA extraction was performed on all Gram-positive strains isolated by the boiling method, following the technique of Olsvick and Strockbine [23] with minor modifications. The isolates were resuspended in BHI broth and incubated in a BOD incubator overnight, and then transferred to 1.5 mL microtubes, centrifuged at 8000 rpm for 4 min, and the supernatant was discarded. They were then resuspended in 200 μL of TE (Tris-Acetate-EDTA, Inc., Sigma-Aldrich MilliporeSigma, Burlington, USA) buffer solution, vortexed, and centrifuged again, with supernatant being discarded and the process repeated three times. At the end of the last wash, the pellet was resuspended, placed in a water bath at 95 °C for 10 min, centrifuged for 20 s at 8000 rpm, and the 450 uL supernatant was then transferred to a 500 uL microtube and stored at −20 °C until use.

### 2.7. Detection of Operon Genes ica (ica A and ica D)

The strains were subjected to the PCR technique as described by Proietti et al. [24], with the primers F: ACTGTTTCGGGGACAAGCAT and R: ATTGAGGCTGTAGGGCGTTG (134 bp) for the gene *ica* A and F: CGTTAATGCCTTCTTTCTTATTGCG and R: ATTAGCGCACATTCGGTGTT (166 bp) for the gene *ica* D. The reaction was performed with 25 μL and with reagents from the PCR kit from Promega Corporation (GoTaq^®^ G2 Master Mix, Inc., Promega Corporation, Madison, Wisconsin, USA) containing 5 μL of Buffer with MgCl2 (5X), 200 μM of deoxyribonucleotide triphosphates (dNTP), 10 μM of each primer, 1 U of Taq polymerase, and 30 ng/μL of DNA (Nanodrop^®^, Inc., NanoDrop Technologies, Wilmington, Delaware, USA) from each sample. The amplification cycle used was (94 °C/3 min, 35 × (94 °C/15 s, 60 °C/20 s, 72 °C/20 s), final extension at 4 °C) performed in a thermocycler (Bioer^®^ Inc., Hangzhou Bioer Technology, Hangzhou, China), and the visualization of the reaction products was performed in 1.5% agarose gel electrophoresis in 0.5X TBE buffer (0.05 mm Tris, 1.25 mm EDTA, and 0.05 M boric acid stained with ethidium bromide), using 10 uL of each sample and 1 uL of 1 Kb molecular size marker (Kasvi^®^, Inc., Kasvi Import and Distribution of Products for Laboratories Ltd., Pinhais, Brazil), with this only in the first well. The gel was visualized under ultraviolet light after the products were stained with Gelred (Invitrogen^®^, Inc., Thermo Fisher Scientific, Waltham, MA, USA). The positive control of the reaction was a strain of *S. pseudintermedius* carrier of the operon genes *ica.*

### 2.8. Statistics

The results regarding identification, antimicrobial susceptibility testing, and phenotypic biofilm production were expressed as a percentage of positivity frequency (%), while the results of genetic analysis were entered into the software BioEstat^®^ version 5.0, to calculate the Kappa coefficient agreement [25].

### 2.9. Limitations

This study has limitations related to its small sample size, operational constraints in the hospital environment, and manual data collection and processing, which limited the number of samples. Microbiological identification was limited to the genus level for some bacteria due to a lack of specialized supplies and equipment and limited access to molecular methods.

## 3. Results

Of the 33 samples collected, 66 isolates were obtained, with 22 isolates from the general areas, 21 isolates from the first collection from the surgical suite, and 23 isolates from the second collection from the surgical suite. Of these, 77% (51/66) were Gram-positive and 23% (15/66) were Gram-negative. The frequency of positivity for the isolated Gram-positive species were, respectively, as follows: *Bacillus* sp. 27% (18/66), *Staphylococcus epidermidis* 17% (11/66), other unidentified species of coagulase-negative staphylococci 15% (10/66), *Staphylococcus pseudintermedius* 12% (8/66), *Staphylococcus aureus* 3% (2/66), and *Enterococcus* sp. 3% (2/66). For Gram-negative bacteria, they were as follows: *Enterobacter* sp. 9% (6/66), *Escherichia coli* 8% (5/66), *Klebsiella pneumoniae* 4% (3/66), and *Acinetobacter* sp. 1.5% (1/66). Table 1 shows the collected locations, the identified species, and their respective resistance profiles and biofilm production capacity through the phenotypic test.

For the general areas, the place with the highest number of isolates was the reception desk with three isolates: *S. pseudintermedius*, *S. epidermidis*, and *E. coli*, while the place with the least isolates was the stethoscope in the triage office, with only one isolate, which was *S. pseudintermedius*. For the surgical suite, the place with the most isolates was the marble bench, which, with the two collections added, totaled seven isolates: two isolates of *S. epidermidis,* two isolates of *K. pneumoniae,* an isolate of coagulase-negative *S.,* and two isolates of *Bacillus* sp., while the place that had the least isolates was the sink, which, with the two collections added and this being a common place for the two surgical rooms of the suite, had no isolates.

**Figure 1:**
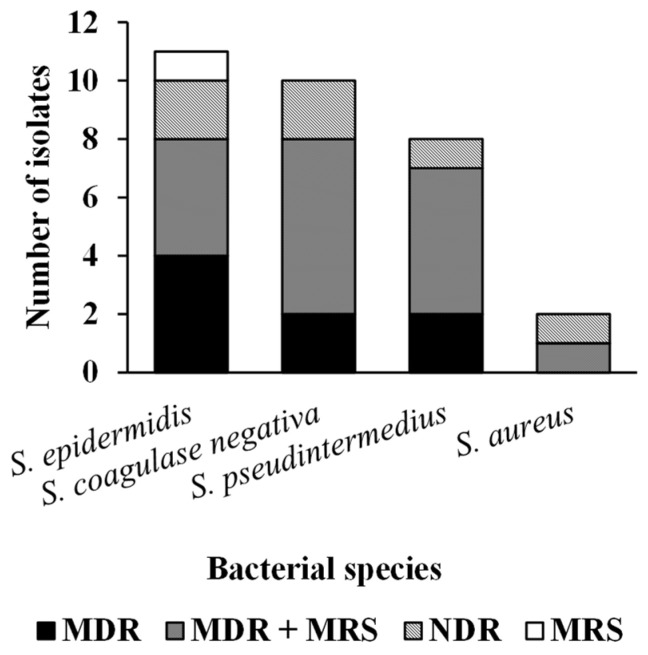
Resistance profiles for isolated species of the genus *Staphylococcus*.

**Figure 2:**
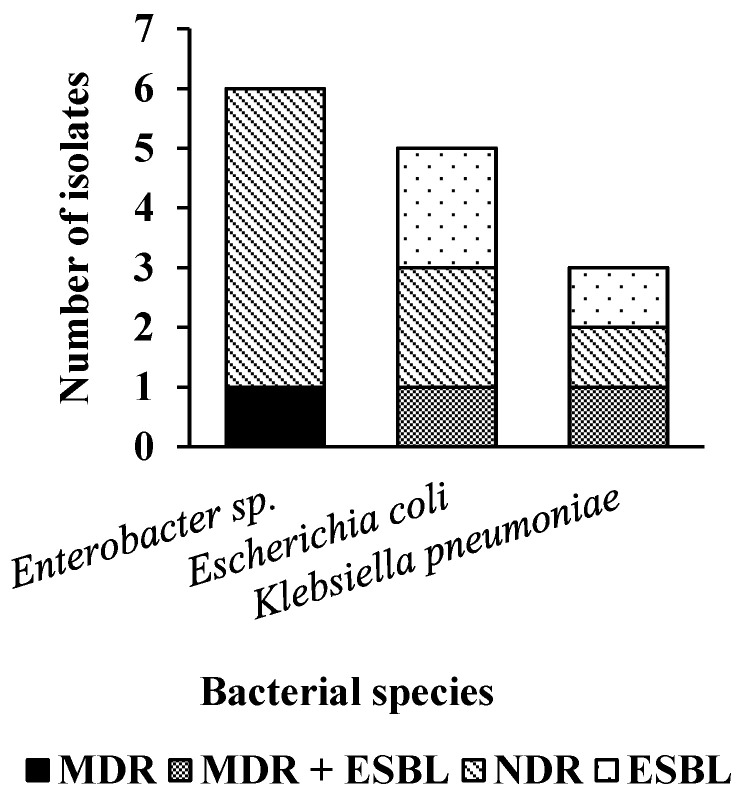
Resistance profiles for isolated species of the genus *Enterobacteriaceae*.

Regarding the antimicrobial susceptibility test (AST), it was possible to observe MDR, ESBL, MRSA, and MRSE resistance patterns and other unidentified species of coagulase-negative staphylococci (MRSCNS). In this study, 44% (29/66) bacteria were MDR, with 90% (26/29) being Gram-positive and 10% (3/29) Gram-negative, and no bacteria classified as XDR or PDR. For ESBL profile, 36% (5/14) samples presented this profile. The only species that did not show resistance to any antimicrobial was the isolate of *Acinetobacter* sp. The following graphs show the resistance profiles found for the species of the genus *Staphylococcus* (Figure 1) and isolated *Enterobacteriaceae* (Figure 2).

For the *Enterococcus* sp., the two isolated samples presented NDR profiles. As for the species of *Bacillus* sp., two samples were MDR and sixteen were NDR.

Biofilm production by the phenotypic test resulted in a total of 57% (38/66) of the isolates capable of forming biofilm. Approximately 79% (30/38) of these forming bacteria were Gram-positive ones, with 12 isolates from *Bacillus* sp., 5 isolates of *S. epidermidis*, 5 isolates of other species of coagulase-negative staphylococci, 5 isolates of *S. pseudintermedius*, 2 isolates of *S. aureus,* and 1 isolate of *Enterococcus* sp. For Gram-negative, 21% (8/38) were biofilm formers, with three isolates of *Enterobacter* sp., three isolates of *E. coli*, one isolate of *K. pneumoniae,* and one isolate of *Acinetobacter* sp. Regarding the classification according to degree of formation, 42% (28/66) of the samples were not biofilm formers, 92% (35/38) were weak formers, and 8% (3/38) were moderate producers, with no strong producer samples (0/38).

Genetic analysis of the operon *ica* (*ica* A and *ica* D) was carried out for the species of *Staphylococcus* sp., with the results being compared with those obtained by phenotypic analysis, which are expressed in Table 2 below:

Phenotypically, 55% (17/31) bacteria were able to produce biofilm, while 45% (14/31) were not. Genotypically, 23% (7/31) were positive only for the gene *ica* D, 19% (6/31) were positive for the gene *ica* A and D, and 58% (18/31) did not present any of the genes researched. When comparing the results, a discrepancy between them could be observed, with the *Kappa* index being −0.0164, with weak agreement between the two parameters analyzed.

## 4. Discussion

Hospital surfaces, equipment, and objects are important reservoirs of micro-organisms and have been extensively studied [26,27,28,29,30,31]. In the present study, 66 bacterial isolates were obtained from various surfaces and objects in a veterinary hospital, with 77% (51/66) being Gram-positive and 23% (15/66) Gram-negative. These findings are consistent with those of Souza et al. [31], who isolated 105 bacteria from surfaces and equipment in a human hospital in Paraná, Brazil, with 84% (88/105) being Gram-positive and 16% (17/105) Gram-negative.

Although most comparative studies are from human hospitals—given the scarcity of data from veterinary facilities—these results suggest a similar pattern of microbial contamination, supporting a One Health perspective. Moreover, the pathogens responsible for healthcare-associated infections (HAIs) in humans are often the same as those found in veterinary contexts [32], which justifies comparisons between these environments. This also reflects the growing concern about antimicrobial resistance (AMR), since similar therapeutic agents are used in both human and veterinary medicine.

The most frequently isolated bacterium in this study was *Bacillus* sp. (27%, 18/66). Similar findings were reported by Palace et al. [28], with 52% (11/21) of isolates being *Bacillus* sp., by Renner and Carvalho [29], who found 15% (6/40) on ICU surfaces, and by Rosa et al. [33], who identified 9.66% (38/392) in ICU aerosols. Although *Bacillus* spp. is not typically associated with HAIs, its ability to form biofilms on hospital surfaces poses a significant challenge, as these biofilms can facilitate the persistence and colonization of other micro-organisms, acting as microbial reservoirs [34].

*Staphylococcus epidermidis* accounted for 17% (11/66) of isolates, and was found on multiple surfaces. Other coagulase-negative *Staphylococcus* species represented 15% (10/66), and were found exclusively in the surgical suite. *S. pseudintermedius* (12%, 8/66) and *S. aureus* (3%, 2/66) were also isolated from general hospital areas. These species are commonly found on hospital equipment such as stethoscopes and bedside rails [35], and on frequently touched surfaces like doorknobs, keyboards, tables, beds, and ICU ventilators [29,36,37,38]. Their presence in animals hospitalized in veterinary settings has been reported with growing frequency [39], highlighting their role as opportunistic pathogens. They are responsible for infections such as pyoderma, otitis externa, urinary tract infections, pneumonia, bacteremia, and, notably, 74% of surgical site infections in veterinary hospitals—of which only 26% respond to standard prophylactic treatment—posing a serious risk to both animal and public health [40].

*Enterococcus* sp. (3%, 2/66) was isolated from two surgical suite rooms. Grosh et al. [41] also found this genus on cage doors, thermometers, and stethoscopes in a veterinary hospital. It has also been identified on surfaces and floors in human hospitals [29,42]. Given its natural presence in the gut microbiota of dogs and cats, contamination likely occurs via fecal shedding. Despite the low incidence found here, this genus warrants monitoring due to its association with hard-to-treat infections [43].

Among Gram-negative isolates, *Enterobacter* sp. (9%, 6/66) was most common, followed by *Escherichia coli* (8%, 5/66) and *Klebsiella pneumoniae* (4%, 3/66), which were found across both general and surgical areas. These Enterobacteriaceae species are part of the normal intestinal flora of humans and animals but are frequently associated with AMR and infections such as urinary tract and bloodstream infections [44]. Previous studies have reported these organisms on floors, waste bins, and bed rails in human hospitals [45,46,47], and on surfaces and equipment in veterinary settings, often with extended-spectrum beta-lactamase (ESBL) or multidrug-resistant (MDR) profiles [27,30]. *Acinetobacter* sp. (1.5%, 1/66) was found in the surgical suite and was sensitive to all antimicrobials tested, consistent with findings by Souza et al. [47], who also reported strains with full susceptibility.

The antimicrobial resistance profiles observed are in line with data from previous studies and highlight the ongoing global threat posed by AMR [27]. This issue is driven by excessive and inappropriate use of antimicrobials for prophylaxis, growth promotion, and export standards, along with poor regulation, lack of oversight, and improper disposal of residues in industrial effluents—all of which contribute to the spread and selection of resistant micro-organisms in the environment [48,49].

Biofilm formation was detected in at least one isolate of each species identified, reinforcing the capacity of these micro-organisms to adhere and persist on hospital surfaces. This is particularly concerning, as biofilms confer greater resistance to antimicrobials and facilitate horizontal gene transfer, exacerbating treatment failures and contributing to increased morbidity and mortality in animals. While most studies on biofilms focus on clinical isolates, few assess their presence on hospital surfaces. However, Silva et al. [50], demonstrated that 93% (13/13) of bacterial isolates from stethoscopes in a general hospital produced biofilms, with 85% (11/13) exhibiting moderate biofilm formation. These findings highlight the risk posed by micro-organisms on abiotic surfaces, comparable to those found in clinical infections.

Our results support the role of biofilms as key virulence and resistance mechanisms, regulated by multiple genetic pathways. This may explain the lack of agreement between phenotypic biofilm production and the presence of *ica* A and *ica* D genes among *Staphylococcus* isolates in our study, reflected by a negative Kappa index (–0.0164) [12]. The iterature indicates that biofilm formation and *ica* operon expression are influenced by environmental and strain-specific factors, including post-transcriptional regulation and suppression under anaerobic or in vitro conditions, which were present in our experimental design [12,50]. Other regulatory systems such as the *agr* ABCD quorum-sensing operon, regulatory RNAs (RNAII, RNAIII), and surface-associated proteins (e.g., Bhp, Aap) can also mediate biofilm production independently of polysaccharide intercellular adhesin (PIA) synthesis, traditionally associated with the *ica* operon [13,51,52].

Although this study focused on detecting *ica* A and *ica* D, we acknowledge the need for broader molecular investigations to elucidate the complex regulatory networks involved in biofilm formation in these bacterial isolates.

## 5. Conclusions

This study identified a number of potentially pathogenic micro-organisms distributed throughout the various areas of the veterinary hospital. Studies of surfaces and objects related to veterinary environments are largely lacking in the literature. Gram-positive bacteria predominated, which is explained by their widespread presence in the human and animal skin microbiota, exhibiting high rates of antimicrobial resistance and the ability to form biofilms. Although the study focused specifically on the presence of the *ica* A and *ica* D genes of the Staphylococcus genus, which are associated with biofilm formation, we recognize that biofilm development is a multifactorial process that may also involve other regulatory systems, such as *agr*, *bhp*, and *aap*. These alternative pathways were not evaluated in this study and represent a significant limitation in the molecular scope of our analysis. The results reinforce concerns about the spread of these micro-organisms in hospital settings and their potential zoonotic potential. They highlight the need for appropriate prevention measures, implementation, and execution of hospital infection control programs in veterinary settings, and the need for new multicenter and longitudinal studies, with increased sample sizes, collection sites, and timeframes to better identify potential reservoirs, prevent outbreaks, and contribute epidemiological data for public and veterinary health. Despite its limitations, this study provides valuable information on the spread of micro-organisms in veterinary hospital settings.

## Figures and Tables

**Table 1:** Collection sites, identified species, resistance profile, and biofilm production in general areas and operating rooms of the veterinary hospital.

Non-Critical Areas (General Areas)				Critical Areas (Operating Room)						
Collection 01				Collection 02				Collection 03		
Locations of Collection	Species Bacterial	Resistance Profile	Biofilm Production	Locations of Collection	Species Bacterial	Resistance Profile	Biofilm Production	Species Bacterial	Resistance Profile	Biofilm Production
Reception desk computer keyboard	*K. pneumoniae* *S. pseudintermedius*	ESBL MDR	No Weak	Heart rate monitor cable pigtails	*S. epidermidis* (S2) *Bacillus* sp. (S2)	MDR MDR	Weak Moderate	*Bacillus* sp. (S1) *Bacillus* sp. (S2) *Enterobacter* sp. (S2)	NDR NDR NDR	No Moderate No
Screening room stethoscope	*S. pseudintermedius*	MDR	No	Heart monitor	*S. epidermidis* (S1) *S. epidermidis* (S2)	MDR/MRSE MDR	No Weak	*Bacillus* sp. (S1) Other species of coagulase-negative staphylococci. (S2) *Enterobacter* sp. (S2)	NDR MDR/MRCNS NDR	No Weak No
Reception desk table	*S. pseudintermedius* *S. epidermidis* *E. coli*	MDR/MRCNS MRSE ESBL	No No Weak	Anesthesia machine (vaporizer)	Other species of coagulase-negative staphylococci. (S2) Other species of coagulase-negative staphylococci. (S2) *S. epidermidis* (S2)	MDR MDR/MRCNS NDR	No Moderate No	Other species of coagulase-negative staphylococci (S1) *Bacillus* sp. (S1) *S. epidermidis* (S2)	NDR NDR MDR	Weak Weak No
Office 02 reception desk	*E. coli* *Bacillus* sp.	ESBL NDR	Weak Weak	Operating table	*S. pseudintermedius* *Acinetobacter* sp. (S2)	NDR NDR	No Weak	*Bacillus* sp. (S1) *Bacillus* sp. (S2)	NDR NDR	No No
Office 02 table for animal screening	*Enterobacter* sp. *S. epidermidis*	NDR MDR/MRSE	No Weak	Thermal mat	No bacterial growth	-	-	*Enterococcus* sp. (S1) *Bacillus* sp. (S2)	NDR NDR	Weak No
Marble counter in the admission room	*Enterobacter* sp. *S. pseudintermedius*	MDR/ESBL MDR/MRCNS	Weak Weak	Table of surgical instruments	*S. epidermidis* (S1) *S. pseudintermedius* (S2)	MDR MDR/MRCNS	No Weak	*S. pseudintermedius* (S2) *Bacillus* sp. (S2)	MDR/MRCNS MDR	Weak Weak
Animal care table in the admission room	*E. coli* *S. aureus*	NDR MDR/MRCNS	No Weak	Marble countertop	*S. epidermidis* (S1) *K. pneumoniae* (S1) Other species of coagulase-negative staphylococci. (S2) *K. pneumoniae* (S2)	MDR/MRSE NDR MDR/MRCNS MDR/ESBL	Weak Weak No No	*Bacillus* sp. (S1) *S. epidermidis* (S1) *Bacillus* sp. (S2)	NDR MDR/MRSE NDR	Weak Weak Weak
Microwave in the admission room	*S. aureus* *Enterobacter* sp.	NDR MDR	Weak Weak	Surgical light	Other species of coagulase-negative staphylococci. (S2)	NDR	Weak	*Bacillus* sp. (S1) *S. epidermidis* (S1) Other species of coagulase-negative staphylococci (S2)	NDR NDR MDR/MRCNS	Weak No No
Animal pen in the admission room	*S. pseudintermedius* *Enterobacter* sp.	MDR/MRCNS NDR	Weak Weak	PPE cabinet surface	Other species of coagulase-negative staphylococci (S2) Other species of coagulase-negative staphylococci (S2)	MDR MDR/MRCNS	No No	No bacterial growth		
Infectious Diseases Animals pen	*Bacillus* sp. *Bacillus* sp.	NDR NDR	Weak No	* Sink tap and basin	No growth Bacterial	-	-	No bacterial growth	-	-
Equipment named preoperative room trachea	*Bacillus* sp. *Bacillus* sp.	NDR NDR	Weak Weak	* Microwave	*E. coli* *E. coli* *Enterococcus* sp.	NDR NDR NDR	No Weak No	Other species of coagulase-negative staphylococci *Bacillus* sp.	MDR/MRCNS NDR	Weak Weak

MDR—Multidrug-resistant; NDR—Does not present multidrug resistance; MRSA—methicillin-resistant Staphylococcus aureus; MRCNS—methicillin-resistant *Staphylococcus pseudintermedius*; MRCNS—methicillin-resistant *Staphylococcus* sp., ESBL—extended spectrum β-lactamase. S1—room 1 of the surgical suite, and S2—room 2 of the surgical suite. * common areas for both rooms of the operating room. PPE: Personal Protective Equipment.

**Table 2:** Comparison between the results of the phenotypic test with crystal violet and the genotypic test of the genes *ica* A and *ica* D for biofilm formation.

Collection 1			Collection 2			Collection 3		
Bacterial Species	Biofilm Phenotypic	Biofilm Genotypic	Bacterial Species	Biofilm Phenotypic	Biofilm Genotypic	Bacterial Species	Biofilm Phenotypic	Biofilm Genotypic
*S. pseudintermedius* *S. pseudintermedius* *S. pseudintermedius* *S. epidermidis* *S. epidermidis* *S. pseudintermedius* *S. aureus* *S. aureus* *S. pseudintermedius*	Weak No No No Weak Weak Weak Weak Weak	No No No No No No No No No	*S. epidermidis* *S. epidermidis* *S. epidermidis* Other species of coagulase-negative staphylococci Other species of coagulase-negative staphylococci *S. epidermidis* *S. pseudintermedius* *S. epidermidis* *S. pseudintermedius* *S. epidermidis* Other species of coagulase-negative staphylococci Other species of coagulase-negative staphylococci Other species of coagulase-negative staphylococci Other species of coagulase-negative staphylococci	Weak No Weak No Moderate No No No Weak Weak No Weak No No	Ica A and D No Ica D Ica D Ica D Ica D Ica D Ica A and D Ica A and D Ica D No Ica D No No	Other species of coagulase-negative staphylococci Other species of coagulase-negative staphylococci *S. epidermidis* *S. pseudintermedius* *S. epidermidis* *S. epidermidis* Other species of coagulase-negative staphylococci Other species of coagulase-negative staphylococci	Weak Weak No Weak Weak No No Weak	No No No No No Ica A and D Ica A and D Ica A and D

## Data Availability

Data is contained within the article.

## Abbreviations

The following abbreviations are used in this manuscript:

MDR	Multidrug-resistant
MRSA	Methicillin-resistant *Staphylococcus aureus*
MRSP	Methicillin-resistant *Staphylococcus pseudintermedius*
MRS	Methicillin-resistant *Staphylococcus* sp.
ESBL	Extended spectrum β-lactamase
NDR	Does not present multidrug resistance
XDR	Extensively drug-resistant
PDR	Pandrug-resistant
HAIs	Healthcare-Associated Infections
EPS	Extracellular polymeric substance
PIA	Polysaccharide intercellular adhesin
ICA	*ica* operon
BHP	Homologous biofilm-associated protein
AAP	Accumulation-associated protein
BHI	Brain Heart Infusion
AST	Antimicrobial susceptibility testing
BrCAST	Brazilian Committee on Antimicrobial Susceptibility Testing
TSB	Tryptic Soy Broth
dNTP	deoxyribonucleotide triphosphates
